# STAT5 and TET2 Cooperate to Regulate FOXP3-TSDR Demethylation in CD4^+^ T Cells of Patients with Colorectal Cancer

**DOI:** 10.1155/2018/6985031

**Published:** 2018-06-14

**Authors:** Hulin Ma, Weishi Gao, Xiaoxia Sun, Weixing Wang

**Affiliations:** ^1^Department of General Surgery, Renmin Hospital of Wuhan University, Wuhan, Hubei, China; ^2^Department of Abdominal Tumor Surgery, Inner Mongolia People's Hospital, Inner Mongolia Autonomous Region, China

## Abstract

The tumor-infiltrating Tregs are linked to colorectal cancer progression and outcome. FOXP3 is regarded as a critical developmental and functional factor for Tregs. FOXP3-TSDR demethylation is required for stable expression of FOXP3 and maintenance of Treg function. In our study, we found specific DNA hypomethylation of FOXP3-TSDR in CD4^+^ T cells from colon tumor tissues as compared with normal colonic tissues. Moreover, we also found that the expression of STAT5 and TET2 was increased in CD4^+^ T cells from colon tumor tissues, and the superfluous STAT5 and TET2 binding to FOXP3-TSDR resulted in DNA hypomethylation. In conclusion, we have demonstrated that excessive amounts of STAT5 may bind more TET2 to the FOXP3-TSDR and upregulate FOXP3 expression via DNA demethylation. Our study improved the mechanism of FOXP3-TSDR hypomethylation in tumor-infiltrating CD4^+^ T cells of CRC patients.

## 1. Introduction

Colorectal cancer (CRC) is the development of cancer in the colon or rectum. It is one of the most common fatal malignancies in the world; globally, more than 1 million people get colorectal cancer every year [1]. The incidence of CRC in China is lower than that in the west countries but has increased in recent years and has become a substantial cancer burden in China, particularly in the more developed areas [2].

Tregs are considered to be a major cell population involved in tumor immune tolerance [3, 4]. The transcription factor forkhead box P3 (FOXP3) is a subfamily member of forkhead transcription factor, which is specifically expressed in CD4^+^CD25^+^ Tregs, and it is regarded as a critical developmental and functional factor for Tregs [5]. Treg-specific demethylated region (TSDR) is a CpG dinucleotide-rich and highly conserved region within the conserved noncoding sequences 2 (CNS2) at the FOXP3 locus [6]. TSDR demethylation is necessary for stable FOXP3 expression and maintenance of the suppressive phenotype for nTregs [7].

Recently, investigators have evaluated that the tumor-infiltrating FOXP3^+^ Tregs are linked to colorectal cancer progression and outcome [8–10]. Zhuo et al. found significantly higher FOXP3-TSDR demethylation rates in tumor sites versus normal sites in patients with CRC, as well as significantly more FOXP3 mRNA expression and higher protein synthesis in tumor tissues [11]. However, the molecular mechanism of FOXP3-TSDR excessive demethylation in patients with CRC has not been fully understood.

Ten-eleven translocation proteins (TET1, TET2, and TET3) catalyze 5-methylcytosine (5mC) conversion to 5-hydroxymethylcytosine (5hmC) to mediate DNA demethylation [12]. It has been found that TET1 and TET2 bind to the FOXP3 CNS2 region in Tregs, suggesting a role for the TET proteins in the maintenance of Tregs identity [13]. Moreover, IL2 can maintain the high level of TET2 during the thymic Treg development, and downregulation of TET2 expression prevents FOXP3-TSDR demethylation [14]. Nevertheless, the molecular mechanism of TET2 targeting regulation of FOXP3-TSDR demethylation is still unclear. STAT5 is an important transcription factor for regulating FOXP3 expression. In the FOXP3 locus, there are multiple STAT5 binding sites, which are located in the 5′-flanking and intron regions including CNS2 [15–17]. However, whether STAT5 participates in the FOXP3-TSDR demethylation process is still unknown.

In this study, we aimed to investigate the correlation between STAT5 and TET2 in demethylation process of FOXP3-TSDR in CD4^+^ T cells of patients with CRC. Our data indicated that STAT5 and TET2 were significantly upregulated in tumor-infiltrating CD4^+^ T cells of CRC. We also provided evidences indicating that overexpressed STAT5 combines with TET2 to form complexes that bind to the FOXP3-TSDR, resulting in excessive demethylation in tumor-infiltrating CD4^+^ T cells. Taking together, our findings reveal that the increased STAT5 and TET2 play an important role in augment of FOXP3 and the pathogenesis of CRC.

## 2. Materials and Methods

### 2.1. Patients and Samples

24 CRC patients have been recruited from the Department of General University of Renmin Hospital of Wuhan University in this study, these patients comprised 16 males and 8 females, ages 46-67 years. This study was approved by the Medical Ethics Committee of Renmin Hospital of Wuhan University, and written informed consent was obtained from all patients. Patients have been diagnosed as CRC according to the histopathological results of the bioptic tissues. Only patients who underwent colectomy for colon cancer without chemotherapy or radiotherapy before surgery were selected, no matter their TNM stage. The characteristics of these patients are shown in Table 1.

Fresh colon tumor tissues and corresponding normal colonic tissues were obtained immediately after surgery. The mononuclear cells were separated from these tissues in the following protocol. In short, the tissue samples were minced and incubated at 37°C for 20 min in Hanks' buffered saline solution (HBSS) containing 2.5% FBS and 1 mM dithiothreitol, and then incubated in 0.75 mM EDTA at 37°C in three rounds of 15 min each, mucus and epithelial cells were removed, respectively. Further, samples were transferred in gentleMACS C tubes (Miltenyi) containing calcium and magnesium supplemented HBSS with 0.5 mg/mL Collagenase IV (Sigma), 50 ng/mL DNAse I (Thermo Fisher), 2% FBS, and 10% BSA. Tissue dissociation was made on a gentleMACS Octo Dissociator with Heaters (Miltenyi). Single cell suspensions were washed with cold HBSS. Then, cells were pelleted through a 40% isotonic Percoll solution and finally centrifuged over a Lympholyte density gradient. CD4^+^ T cells were further isolated from mononuclear cells using human CD4 beads (Miltenyi) according to protocols provided by the manufacturer.

### 2.2. RNA Isolation and Real-Time PCR

Total RNA extraction in CD4^+^ T cells was performed with TRIZOL (Invitrogen). cDNA was synthesized according to the instructions in the SuperScript II reverse transcriptase (Invitrogen). mRNA expression was assessed by real-time PCR using the 7500 Real-Time PCR Systems (Applied Biosystems). The mRNA levels of various genes were calculated after normalizing with GAPDH, and the 2^−ΔΔCt^ method was used for analyzing target gene expression level. Primers are listed in Table 2.

### 2.3. Western Blot Analysis

CD4^+^ T cells were lysed in 1 ml of lysis buffer containing protease inhibitor (Thermo Scientific) at 4°C. Lysates were centrifuged for 15 min at 14,000*g* at 4°C, and protein concentration was determined by Bradford protein assay (Thermo Scientific). Soluble lysates were loaded in each lane and separated by sodium dodecyl sulfate-polyacrylamide gel electrophoresis and transferred to polyvinylidene difluoride membranes (Millipore). Membranes were blocked with 5% nonfat dry milk in Tris-buffered saline containing 0.1% Tween-20 buffer (TBST) for 1 hr, washed with TBST, and incubated with primary antibodies. Antibodies include anti-STAT5 (ab126832), anti-TET2 (ab94580), anti-FOXP3 (ab10901), and anti-GAPDH (ab181602); all antibodies were obtained from Abcam Company. Blots were rinsed with TBST and subsequently incubated with horseradish peroxidase-conjugated secondary antibodies for 1 hr at room temperature. The intensity of the identified bands was quantified using Quantity One software (Bio-Rad).

### 2.4. Genomic DNA Extraction and Bisulfite Sequencing

We followed the method of Xu et al. [18]: genomic DNA was isolated from CD4^+^ T cells using the QIAamp DNA Mini Kit (Qiagen). Bisulfite conversion was performed using the EpiTect Bisulfite Kit (Qiagen). The FOXP3-TSDR was amplified by PCR. The PCR products were next subcloned into a pGEM-T vector (Promega, WI, USA). Ten independent clones were sequenced for each of the amplified fragments. Primers used were as follows: Forward1: 5′TAGAGGGTTAATGGGTAGTTATGT3′ and Reverse1: 5′GAAATCTCCCACTTTTTAAACCTCTA3′; Forward2: 5′TAGAGGTTTAAAAAGTGGGAGATTT3′ and Reverse2: 5′TAATACTATCCTTTAAAATCTCTA3′.

### 2.5. Chromatin Immunoprecipitation (ChIP)

ChIP analysis were performed according to the instructions provided by the ChIP assay kit (Millipore). To crosslink proteins to DNA, add 27 *μ*l of 37% formaldehyde to each 1 ml CD4^+^ T cells. Swirl briefly to mix and incubate 10 min at room temperature. Add 100 *μ*l of 10x glycine to each sample to quench the formaldehyde. Pellet cells by centrifugation at 1500 rpm 5 min, wash pellet two times with 20 ml ice-cold PBS, and lyse. Pellet and resuspended lysates reduce DNA into 500–1000 base pair fragments by sonication. Add 2 *μ*g anti-STAT5 (#94205), or anti-TET2 (#18950), or control rabbit IgG (#2729) and incubate overnight at 4°C with rotation; all antibodies were obtained from Cell Signaling. Chromatin was precipitated with protein A agarose beads for 1 hr. The immune complexes were washed and eluted in 100 *μ*l of TE containing 0.5% SDS and 200 *μ*g/ml proteinase K. Precipitated DNA was further purified with spin columns before amplifying target DNA by real-time PCR. Several pairs of primers used to detect the enrichment of FOXP3-TSDR, which reflect the combining capacity of STAT5 and TET2. Primers used were as follows: Distal promoter forward: GCATAGGCGGTGAGATGAAG and Distal promoter reverse: TTCTTCATCATGGATTTCGCA, CNS1 forward: TAGAGCTTCAGATTCTCTTTCT and CNS1 reverse: GAGATAATAGGGCTCATGAGA, FOXP3-TSDR forward: GATGACGTACAGCGAATTGAG and FOXP3-TSDR reverse: TGTAGCCATTCCACTTTTCC.

### 2.6. Coimmunoprecipitation

We followed the method of Hou et al. [19]: nuclear proteins from CD4^+^ T cells were extracted using the CelLytic™ NuCLEAR™ extraction kit (Sigma-Aldrich). Nuclear extracts were incubated overnight with anti-STAT5 (#94205), or anti-TET2 (#18950), or control rabbit IgG (#2729) at 4°C. Add 30 *μ*l of ChIP-Grade protein A/G PLUS-agarose beads (Millipore) to each IP reaction and incubate for 2 hr at 4°C with rotation. Pellet protein A/G PLUS-agarose beads by brief 1 min centrifugation at 6000 rpm and remove supernatant. Wash precipitated complex 3 times. Elute proteins from the solid support using SDS sample loading buffer. Analyze complexes by Western blot.

### 2.7. Transfection of CD4^+^ T Cells

CD4^+^ T cells were transfected using the human T cell Nucleofector kit and Amaxa Nucleofector (Lonza). pCMV6-TET2 (SC315236) was obtained from OriGene and siRNA-STAT5 (13MR0030A) was obtained from Invitrogen (Interference sequence: TGCTCATTCAGAATCTGAAGC). We followed the method of Xu et al. [18]: first, CD4^+^ T cells were harvested and resuspended in 100 ul human T cell nucleofector solution, and then the cell suspension was mixed with siRNA-STAT5, pCMV6-TET2, or negative control. The mix was electrotransfected using Nucleofector program V-024 in the Amaxa Nucleofector. The transfected cells were cultured in human T cell culture medium. After 48 hours, the cells were collected.

### 2.8. Statistical Analysis

The paired-samples *t*-test for equality of means was used to compare values. *p* values of less than 0.05 were considered statistically significant. All analyses were performed with SPSS version 16.0 software.

## 3. Results

### 3.1. DNA Methylation of FOXP3-TSDR in Colon Tumor Tissues and Normal Colonic Tissues

**Figure 1:**
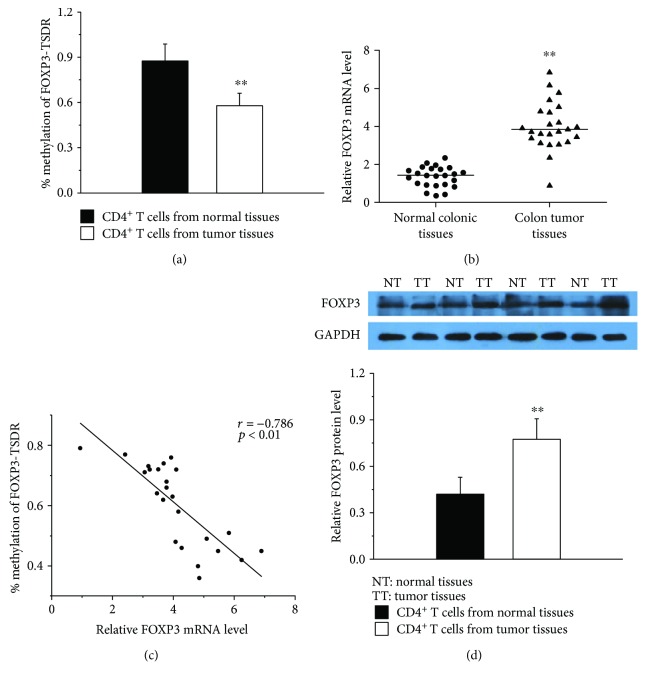
DNA methylation of FOXP3-TSDR in colon tumor tissues and normal colonic tissues. (a) Average methylation status of FOXP3-TSDR in CD4^+^ T cells from colon tumor tissues (*n* = 24) and normal colonic tissues (*n* = 24). (b) Relative FOXP3 mRNA levels in CD4^+^ T cells from colon tumor tissues (*n* = 24) and normal colonic tissues (*n* = 24) normalized to GAPDH. (c) The correlation between methylation levels of FOXP3-TSDR and its mRNA levels (*r* = −0.786, *p* < 0.01, *n* = 24). (d, top) Representative western blot results for FOXP3 in CD4^+^ T cells from colon tumor tissues (*n* = 24) and normal colonic tissues (*n* = 24). GAPDH was used for normalization. (d, bottom) Quantitative analysis of the band intensities for FOXP3 protein levels normalized by GAPDH (^∗∗^*p* < 0.01).

**Figure 2:**
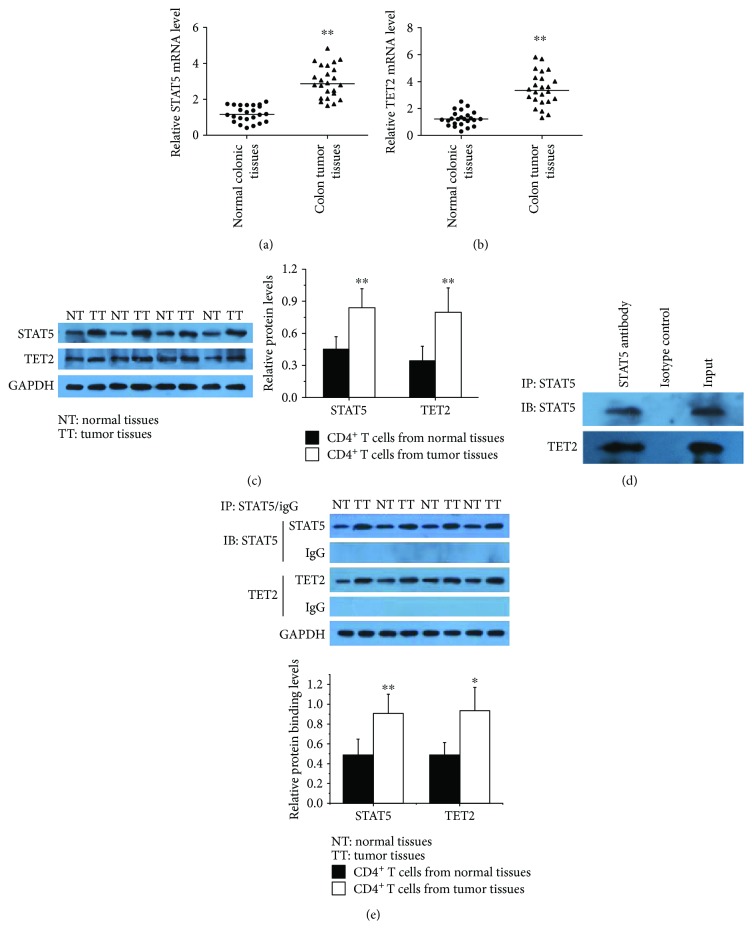
The interrelationship between STAT5 and TET2 in CRC CD4^+^ T cells. (a, b) Relative STAT5 and TET2 mRNA levels in CD4^+^ T cells from colon tumor tissues (*n* = 24) and normal colonic tissues (*n* = 24) normalized to GAPDH. (c, left) Representative Western blot results for STAT5 and TET2 in CD4^+^ T cells from colon tumor tissues (*n* = 24) and normal colonic tissues (*n* = 24). GAPDH was used for normalization. (c, right) Quantitative analysis of the band intensities for STAT5 and TET2 protein levels normalized by GAPDH. (d) Coimmunoprecipitation using anti-STAT5 in CD4^+^ T cells from colon tumor tissues; detection of the combination of STAT5 and TET2 by Western blot. The same experiments were repeated 3 times. (e) Coimmunoprecipitation using anti-STAT5 in CD4^+^ T cells from colon tumor tissues (*n* = 6) and normal colonic tissues (*n* = 6); detection of the binding levels of STAT5 and TET2 by Western blot (^∗^*p* < 0.05, ^∗∗^*p* < 0.01).

To investigate whether the FOXP3-TSDR was aberrantly DNA methylated in CD4^+^ T cells from colon tumor tissues, we did bisulfate sequencing analysis for TSDR. The FOXP3-TSDR was found to be significantly hypomethylated in CD4^+^ T cells from colon tumor tissues as compared with normal colonic tissues (Figure 1(a)). Further, we measured the expression of FOXP3 in colon tumor tissues and normal colonic tissues. The results showed that FOXP3 mRNA (Figure 1(b)) and protein levels (Figure 1(d)) were sharply increased in CD4^+^ T cells from colon tumor tissues. Further, we found that the FOXP3-TSDR DNA methylation was negatively correlated with its mRNA level through the correlation analysis (Figure 2(c)). Together, our results suggest that the abnormally reduced Foxp3-TSDR methylation may be an important factor to increase the expression of FOXP3 in CD4^+^ T cells of CRC patients.

### 3.2. The Interrelationship between STAT5 and TET2 in CRC CD4^+^ T Cells

We measured the mRNA and protein expression levels of STAT5 and TET2 in CD4^+^ T cells from colon tumor tissues coupled with corresponding normal colonic tissues. The results from real-time PCR showed that STAT5 and TET2 expression was significantly upregulated in tumor tissues compared with normal tissues (Figures 2(a) and 2(b)). Western blot further confirmed the elevation of STAT5 and TET2 (Figure 2(c)).

In order to validate the interaction between STAT5 and TET2, we performed coimmunoprecipitation using anti-STAT5 in CD4^+^ T cells from colon tumor tissues. The results showed that STAT5 could combine with TET2 to form a complex (Figure 2(d)), and more complexes were found in tumor tissues compared with normal tissues (Figure 2(e)).

### 3.3. TET2 Binds to the FOXP3-TSDR and Regulates Its DNA Methylation via STAT5

**Figure 3:**
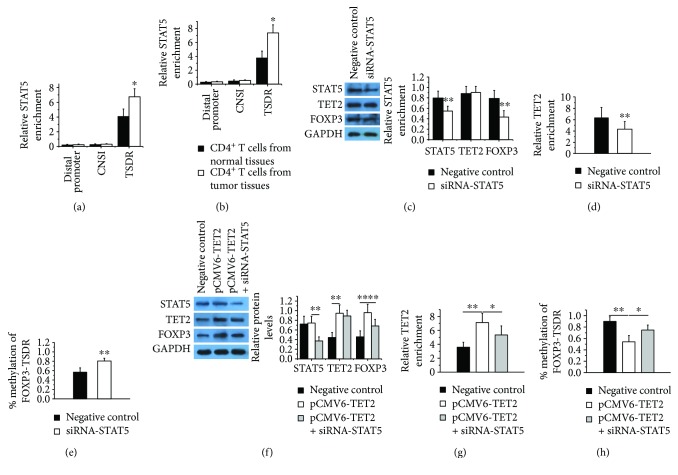
TET2 binds to the FOXP3-TSDR and regulates its DNA methylation via STAT5. (a-b) ChIP-qPCR analysis of the enrichment of STAT5 (a) and TET2 (b) in distal promoter, CNS1, and TSDR of FOXP3 in CD4^+^ T cells from colon tumor tissues (*n* = 12) and normal colonic tissues (*n* = 12). (c, left) Representative Western blot results for STAT5, TET2, and FOXP3 in CD4^+^ T cells from colon tumor tissues after interference with STAT5 expression. GAPDH was used for normalization. (c, right) Quantitative analysis of the band intensities for STAT5, TET2, and FOXP3 protein levels normalized by GAPDH. The same experiments were repeated 3 times. (d) ChIP-qPCR analysis of the enrichment of TET2 in the FOXP3-TSDR in CD4^+^ T cells from colon tumor tissues after interference with STAT5 expression. The same experiments were repeated 3 times. (e) DNA methylation of FOXP3-TSDR in CD4^+^ T cells from colon tumor tissues after interference with STAT5 expression. The same experiments were repeated 3 times. (f, left) Representative Western blot results for STAT5, TET2, and FOXP3 in CD4^+^ T cells from normal human donors after overexpression of TET2 and inhibition of STAT5. GAPDH was used for normalization. (f, right) Quantitative analysis of the band intensities for STAT5, TET2, and FOXP3 protein levels normalized by GAPDH. The same experiments were repeated 3 times. (g) ChIP-qPCR analysis of the enrichment of TET2 in the FOXP3-TSDR in CD4^+^ T cells from normal human donors after overexpression of TET2 and inhibition of STAT5. The same experiments were repeated 3 times. (h) DNA methylation of FOXP3-TSDR in CD4^+^ T cells from normal human donors after overexpression of TET2 and inhibition of STAT5. The same experiments were repeated 3 times (^∗^*p* < 0.05, ^∗∗^*p* < 0.01).

To confirm that the abnormally increased STAT5 and TET2 bind more strongly to FOXP3-TSDR in CD4^+^ T cells from colon tumor tissues compared with which from normal tissues, we performed ChIP-qPCR analysis using anti-STAT5 and TET2 antibody. As shown in Figures 3(a) and 3(b), CRC patients showed stronger binding of STAT5 and TET2 to FOXP3-TSDR in tumor tissues compared with normal tissues.

Further, we suppressed the expression of STAT5 by transfection of siRNA-STAT5 and detected the levels of TET2 binding to FOXP3-TDSR in CD4^+^ T cells from tumor tissues. The result showed that the expression of STAT5 and FOXP3 (Figure 3(c)) and the binding level of TET2 in FOXP3-TSDR (Figure 3(d)) were decreased obviously, while the FOXP3-TSDR methylation (Figure 3(e)) was increased significantly after interference with STAT5 expression. In addition, we upregulated the expression of TET2 with or without STAT5 inhibition in CD4^+^ T cells from normal human donors. We found that overexpression of TET2 resulted in a significant increase in the combination of FOXP3-TSDR (Figure 3(g)), obviously reducing the DNA methylation of TSDR (Figure 3(h)), and upregulating the expression of FOXP3 (Figure 3(f)). Interference with STAT5 expression inhibits the binding of TET2 to FOXP3-TSDR (Figure 3(g)) and partly reverses the reduced DNA methylation of TSDR (Figure 3(h)) and the increased FOXP3 expression caused by TET2 overexpression (Figure 3(f)). Overall, these data suggest that STAT5 may be a key factor in the process of TET2-mediated demethylation of FOXP3-TSDR; excessive amounts of STAT5 may bind more TET2 to the FOXP3-TSDR and upregulate FOXP3 expression via DNA demethylation.

## 4. Discussion

Numerous studies have verified an increase of Tregs in the circulation, draining lymph nodes, and at the tumor sites of patients with malignancy, including breast, lung, pancreas, and skin cancers [8, 20–24]. Colorectal cancer is one of the most common cancers worldwide and a major cause of cancer-related death [25]. Therefore, it is meaningful to explore the Tregs and their possible role in CRC, as well as the potential significance in the targeted therapeutics. For CRC patients, increased numbers of Tregs had been shown in peripheral blood, tumor-draining lymph node, and tumor site, and these Tregs could actively migrate to the site of immune activity [8, 26, 27]. Moreover, Salama et al. found that the high FOXP3^+^ Tregs density in normal colonic mucosa of CRC patients was associated with worse prognosis [9]. Liu et al. demonstrated that colorectal cancer tissues overexpressed FOXP3 compared with normal colorectal tissues. Similarly, the proportion of FOXP3^+^ Tregs in the peripheral blood was higher in CRC patients than in controls. The expression of FOXP3 was positively correlated with Dukes staging and lymph node metastasis. Thus, the excess expression of FOXP3 may be a key factor in CRC pathological process; however, the molecular mechanism of FOXP3 overexpression remains unclear.

STAT5 is necessary and sufficient for Treg development. Specifically, T cell specific deletion of STAT5 prevents Treg development. TGF-*β* signaling pathway can activate STAT5, mediate it binding to the CNS2 region of FOXP3, and promote transcription of FOXP3, but the mechanism is unknown [17]. Ichiyama et al. found that TET2 could promote DNA demethylation and activation of cytokine gene expression in T cells [28]. Nair and Oh through the studies on experimental animals found that downregulation of TET2 prevents FOXP3-TSDR demethylation in IL2 deficient regulatory T cells [14]. In combination with their research, we speculate that TET2 may participate in the process of FOXP3-TSDR demethylation via STAT5 guidance. Our study found significant hypomethylation of FOXP3-TSDR in tumor-infiltrating CD4^+^ T cells of CRC patients, which is consistent with the results of other investigators [11]. Furthermore, we also found that the expression of STAT5 and TET2 were increased in CD4^+^ T cells from tumor tissues, and the massive STAT5 binds more TET2 to the FOXP3-TSDR and upregulates FOXP3 expression. This provides an explanation for the molecular mechanism of STAT5 and TET2 upregulation of FOXP3 expression.

In conclusion, we demonstrated that the excessive expression of FOXP3 in tumor-infiltrating CD4^+^ T cells of CRC patients is caused, at least in part, by the upregulation of STAT5 and TET2. The unbalanced TET2 may bind to the FOXP3-TSDR overly via superfluous STAT5, thereby contributing to DNA demethylation and mRNA transcription. Thus, our study improved the mechanism of FOXP3-TSDR hypomethylation in CD4^+^ T cells of CRC patients and provided a novel therapeutic target of CRC.

## Figures and Tables

**Table 1:** Clinical characteristics of all CRC patients.

Clinical characteristics	*N* = 24
Median age	56 ± 6
Sex	
Male	16
Female	8
Stage (AJCC)	
I	3 (12.5%)
II	6 (25%)
III	11 (45.8%)
IV	4 (16.7%)
Tumor location	
Cecum	1 (4.2%)
Ascending colon	4 (16.7%)
Transverse colon	3 (12.5%)
Descending colon	5 (20.8%)
Sigmoid colon	11 (45.8%)

**Table 2:** Primer sequences for real-time PCR.

	Forward primer	Reverse primer
FOXP3	GAGAAGCTGAGTGCCATGCA	AGAGCCCTTGTCGGATGAT
STAT5	GCCGAGAAGCACCAGAAGACC	CGGCCAGCATCTCCTCCA
TET2	AGGCTAAACAGTTGGCAGA	GGTGGAATAGAAGTTCATAG
GAPDH	AAGAGCTACGAGCTGCCTGAC	ATGGCCCAGCGGATGAG
